# Impact of mentorship on WHO-AFRO Strengthening Laboratory Quality Improvement Process Towards Accreditation (SLIPTA)

**DOI:** 10.4102/ajlm.v1i1.6

**Published:** 2012-02-16

**Authors:** Talkmore Maruta, David Motebang, Lebina Mathabo, Philip J. Rotz, Joseph Wanyoike, Trevor Peter

**Affiliations:** 1 Clinton Health Access Initiative, Maseru, Lesotho; 2 Ministry of Health and Social Welfare, Lesotho

## Abstract

**Background:**

The improvment of the quality of testing services in public laboratories is a high priority in many countries. Consequently, initiatives to train laboratory staff on quality management are being implemented, for example, the World Health Organization Regional Headquarters for Africa (WHO-AFRO) Strengthening Laboratory Management Towards Accreditation (SLMTA). Mentorship may be an effective way to augment these efforts.

**Methods:**

Mentorship was implemented at four hospital laboratories in Lesotho, three districts and one central laboratory, between June 2009 and December 2010. The mentorship model that was implemented had the mentor fully embedded within the operations of each of the laboratories. It was delivered in a series of two mentoring engagements of six and four week initial and follow-up visits respectively. In total, each laboratory received 10 weeks mentorship that was separated by 6–8 weeks. Quality improvements were measured at baseline and at intervals during the mentorship using the WHO-AFRO Strengthening Laboratory Quality Improvement Process Towards Accreditation (SLIPTA) checklist and scoring system.

**Results:**

At the beginning of the mentorship, all laboratories were at the SLIPTA zero star rating. After the initial six weeks of mentorship, two of the three district laboratories had improved from zero to one (out of five) star although the difference between their baseline (107.7) and the end of the six weeks (136.3) average scores was not statistically significant (*p* = 0.25). After 10 weeks of mentorship there was a significant improvement in average scores (182.3; *p* = 0.034) with one laboratory achieving WHO-AFRO three out of a possible five star status and the two remaining laboratories achieving a two star status. At Queen Elizabeth II (QE II) Central Laboratory, the average baseline score was 44%, measured using a section-specific checklist. There was a significant improvement by five weeks (57.2%; *p* = 0.021).

**Conclusion:**

The mentorship programme in this study resulted in significant measurable improvements towards preparation for the WHO-AFRO SLIPTA process in less than six months. We recommend that mentorship be incorporated into laboratory quality improvement and management training programmes such as SLMTA, in order to accelerate the progress of laboratories towards achieving accreditation.

## Introduction

Clinical laboratories form the foundation of evidence-based patient treatment and care, and are a fundamental component of disease surveillance, diagnosis and monitoring at every level of the health care system.^1^ In many low-resource settings, including many African countries, laboratory services have suffered from inattention and chronic under-development. However, in recent years, Ministries of Health have increasingly prioritised the quality of testing services by implementing quality management systems and building quality improvement activities into laboratory service work plans.^2,3,4^ In the African region, the vital importance of laboratories in public health surveillance is part of the framework for the World Health Organization Regional Headquarters for Africa’s (WHO-AFRO) Integrated Disease Surveillance and Response (IDSR) strategies that are adopted in nearly all African countries.

In response to these goals, in 2009 WHO AFRO established the WHO-AFRO Laboratory Accreditation Process^5^ and laboratory management training programmes such as Strengthening Laboratory Management Towards Accreditation (SLMTA^1^), and supported the launch of the African Society for Laboratory Medicine in 2011.^6^ The WHO-AFRO Laboratory Accreditation process was subsequently replaced by the WHO-AFRO Strengthening Laboratory Quality Improvement Process towards Accreditation (SLIPTA) in 2011.

Laboratory management training has been identified as one of the six building blocks in the implementation of a quality management system.^3^ However, pre-existing training programmes failed to result in measurable changes in laboratory practices.^1^ Yao and others identified three key limitations to existing laboratory training programmes, (1) curriculum content, (2) lack of follow up of trainees to assist with application of knowledge into practice and (3) training focusing more on theory around generic management topics and less on practical aspects that can lead to direct implementation.

Establishing well-structured laboratory mentorship programmes has been suggested as a means of accelerating a laboratory’s path toward accreditation.^3^

Various methods of laboratory mentorship have been described and implemented in both developing and developed countries.^7,8,9^ Often mentorship is conducted over either short (less than one week) or long (six months to one year or longer) time periods. We believe that these mentorship models often do not achieve the desired impact. Spending shorter periods of time in the laboratory does not enable the mentor to better understand the rhythms, patterns, practices and personalities of the laboratory in order to foster positive change in process and behaviour. Long term continuous mentorship does not provide the opportunity to assess how well a laboratory is able to sustain or even extend quality improvement in the absence of the mentor.

The purpose of this study was to pilot test a model of laboratory mentorship. A two step 10 week mentorship model was developed in Lesotho in order to address the aforementioned limitations and deliver high-impact mentoring that supports the requirements of the ISO 15189 laboratory quality standard and helps prepare laboratories for the WHO-AFRO SLIPTA process. The purpose of this study was to measure the improvement in quality systems of laboratories receiving this model of mentorship.

## Methods

### Mentorship sites

Mentorship was implemented at four laboratories in Lesotho over an 18 month period between June 2009 and December 2010 (see Table 1 for the profile of the laboratories). Three of the laboratories were Mafeteng, Motebang and Scott hospital laboratories. Scott is a Christian Health Association of Lesotho (CHAL) hospital laboratory and the other two are owned by the government. The fourth facility was Lesotho’s QE II Central Laboratory, located at Queen Elizabeth II Hospital in the capital, Maseru. The district laboratories typically had three modestly-sized rooms dedicated to testing and a small store room. These laboratories were comprised of the following sections: chemistry, haematology, CD4+ T-cell count and TB microscopy. These sections had automated analysers for chemistry, haematology, and CD4 + T-cell count and typically performed tests such as liver function tests, creatinine, CD4 count, full blood count, malaria smears, blood grouping and cross-match, TB microscopy, and a small range of serology rapid tests. One of the three district laboratories had microbiology with culture. The central laboratory had one large room compartmentalised into chemistry, CD4+ T-cell count, haematology, cytology, blood transfusion and histology. At the time of mentorship, all four laboratories used a paper based laboratory information system.

**TABLE 1:** Profiles of the four laboratories where the mentorship model was implemented.

Characteristic	Mafeteng	Scott	Motebang	Queen Elizabeth II Central Laboratory
Level	District	District	District	Central Laboratory
Affiliation	Government	CHAL	Government	Government
Layout	2 testing rooms patient waiting room sample reception area storeroom laboratory office	3 testing rooms sample reception area store room laboratory office	3 testing rooms sample reception area store room laboratory office	1 large testing room laboratory office
Staff	2 technologists^†^ 2 TB microscopists 1 bike driver 1 cleaner	3 technologists 1 laboratory aide^‡^ 1 TB microscopist^†^ 1 bike rider	7 technologists 2 microscopists 2 bike rider	16 technologists 2 bike riders
Sections	Chemistry haematology CD4 Blood bank serology TB microscopy urinalysis	Chemistry haematology CD4 Blood bank hserology TB microscopy urinalysis	Chemistry haematology CD4 Blood bank serology TB microscopy urinalysis	CD4 haematology chemistry cytology
Volume of testing (samples/month)	900–1000	1000–2000	2000–3000	3000–4000
Distance from capital city (km)	~100	~50	~200	In capital city

CHAL, Christian Health Association of Lesotho.

† Technologists hold diploma from local training institute.

‡ Laboratory aides trained 100% on the bench. Microscopists perform TB microscopy only.

During the mentorship period, Mafeteng had three technologists and two microscopists, Motebang seven technologists and two microscopists and Scott had four technologists, one laboratory aide and one microscopist. The staff had three-year diploma qualifications from the local National Health Training College (NHTC). The central laboratory had 16 staff members, consisting of 13 diploma-holders and three BSc degree-holders, of whom three were supervisory staff. None of the laboratories had administration staff dedicated to the laboratory; therefore technologists performed their own data entries.

During the period of mentorship, no other improvement initiatives were undertaken at the laboratories besides the routine six monthly supervisory check-in visits by the Ministry of Health laboratory representative. Typically these were at most 2–3 hour supervisory visits by the National Quality Officers from the quality assurance unit of Ministry of Health Laboratory Directorate.

### Mentor qualification and experience

The laboratory mentor was an experienced laboratorian from the southern African region and had significant experience in quality systems building and training. The mentor was a trained medical laboratory scientist with a four year degree in BSc Medical Laboratory Sciences. At the start of the mentorship, the mentor had eight years of laboratory working experience, with two of these as a trainer in laboratory quality management systems. The mentor had previously worked in a reference laboratory that was preparing for accreditation by the South African National Association of Standards (SANAS).

### Mentorship model

We will now present the mentorship programme characteristics under subheadings.

#### Facility-based approach

To foster a team approach to quality, the mentorship model employed a facility-based approach. The mentor did not focus on specific individuals (e.g. the laboratory supervisor) but rather worked with all laboratory staff, including supervisors, technologists, microscopists and sample transporters. The mentor was embedded within the operations of the laboratory in order to understand its processes, challenges, and the strengths and capabilities of the staff. The mentor worked alongside the laboratory staff as an experienced peer.

#### Time allocation per laboratory

**FIGURE 1:**
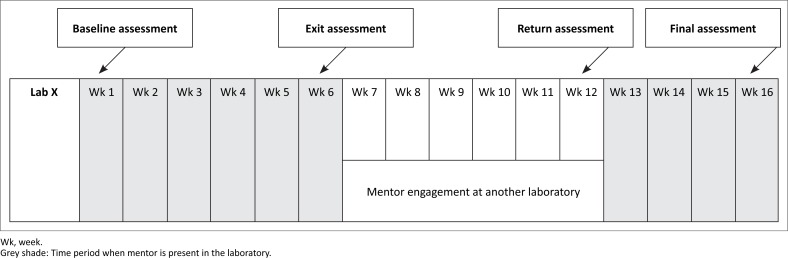
Schematic representation of the 10 weeks of mentorship split into two blocks of six and four weeks with six to eight weeks in between.

The mentorship was designed to ensure a significant amount of time with the mentor embedded within the laboratory in a series of mentoring engagements. Each laboratory received a total of 10 weeks of full time, on-site mentorship. The time was divided between an initial six-week mentoring engagement, a break of between six and eight weeks, and a subsequent four week engagement (Figure 1). The initial six week engagement commenced with a baseline assessment using the WHO-AFRO SLIPTA checklist. The mentor then used the findings of this assessment to determine priority areas for quality improvement in the laboratory. During the initial six-week engagement the mentor worked alongside the laboratory staff to help them address the nonconformities revealed in the assessment. A second measurement with the same checklist was administered at the conclusion of the initial six-week engagement. The 6–8 week gap between the two mentoring engagements was purposely built into the model to provide an opportunity to observe how the laboratory functioned on its own after the establishment of the quality initiatives with the mentor. A third assessment with the WHO-AFRO SLIPTA checklist was performed at the conclusion of this gap period to determine laboratory progress or regress and inform the work plan for the second mentoring engagement. During this second engagement, which lasted four weeks, the performance gains that were already realised were reinforced and areas of continuing concern were pointedly addressed. At the conclusion of the second mentoring engagement the laboratory was again evaluated with the WHO-AFRO SLIPTA checklist.

#### Structured mentorship

To ensure an approach that could be standardised and scaled-up across laboratories, standard laboratory action plans and mentor schedules were formulated for all laboratories. The action plans were formulated following assessments using the WHO-AFRO SLIPTA checklist. A summary of assessment findings was formulated by the mentor (see example in Table 2) and discussed with the laboratory staff before a laboratory action plan was jointly formulated (see example in Table 3). The laboratory action plan consisted of a list of activities to be done, the responsible person, timeline, the signature of the responsible person and the review dates. These became the working documents for the laboratory and defined its improvement path.

**TABLE 2:** Example summary of assessment findings table formulated after each assessment to be used by the laboratory and mentor to generate action plans.

Checklist section	Item	Observation or conformity or nonconformity	15189 Clause	Action or comment
General information	Cleaners and drivers	Training records for cleaners could not be verified. Training of drivers also needs to be updated. Refer to Page 8 WHO SLIPTA checklist.	5.2.10. Work areas to be kept clean and measures shall be taken to ensure good housekeeping. Special procedures and training for personnel could be necessary to that end.	Mentor to assist with design of Bio-Safety Training with Safety Officer.
1.0 Documents and records	Documents and record control	SOPs have effective date but there is no indication of date of retrieval from the system. Refer to Question 1.8 of the WHO SLIPTA checklist.	4.3.2 (a, d) Procedure shall be adopted for review and approval of documents.	QA Office to review document header. Supervisor to make follow ups on progress of the new document control/SOP writing SOP being drafted.

**TABLE 3:** Example of laboratory action plan derived from the summary of assessment findings.

Action item	Responsible persons	Timeline	Signature^†^	Review after due date		
				Review 1^†^	Review 2^†^	Review 3^†^
QA Officer to review document control header	QA Officer	30 June				

† The person assigned the task has to sign as an indication of responsibility of the task. After each due date, the progress of the action item is reviewed and status updated under review 1 to 3.

To allow prioritisation of tasks, streamlining and focusing mentor activities, the mentor also formulated a ’mentor schedule‘ based on the summary of each assessment finding and the laboratory action plan. The mentor schedules listed by weeks the activities that the mentor would focus on to help the laboratory implement its action plan and hence resolve its nonconformities. If the laboratory did not meet requirements of the checklist this was considered as a nonconformity. For example, if the checklist required that the laboratory perform routine stock counts and this requirement was not met, this was considered a nonconformity. The mentor schedule prioritised the resolution of nonconformities that would build a foundation for resolving the other nonconformities during and beyond mentorship. For example, documentation is a priority for any of the subsequent quality improvements, hence this should appear early in the mentorship schedule. Another example would be investigation and documenting corrective actions; the mentor should schedule this early on in order to allow the laboratory to begin implementation under the guidance of the mentor and then continue independently.

### Mentoring methods

Whilst on site, the mentor employed a number of methods and techniques to implement the aforementioned action plans and scheduled activities. The methods will be described in the next section

#### Side-by-side mentoring

The mentor was part of the daily routines of the laboratory and provided coaching whilst working side by side with the laboratory staff, such as coaching on how to perform internal quality controls, calibration, plotting and reviewing of Levy-Jennings (L-J) charts for CD4+ T-cell count testing. During this time the mentor demonstrated a strong work ethic, efficient and professional job performance, and dedication to quality whilst conducting testing alongside the laboratory staff. This enabled the mentor to intimately understand the laboratory and teach by example, and targeted on-the-bench interventions.

#### Targeted mentoring

Special mentoring emphasis was also given to laboratory staff with greater levels of responsibility and specific duties within the laboratory, for example laboratory technical staff assigned to the roles of supervisor, quality officer, safety officer and inventory officer. These individuals received direct mentorship on their specific duties.

#### Group discussions

Discussions on specific topics were done with small groups either at a section level (for example haematology) or for a small team assigned to specific tasks. Topics for discussion were drawn from nonconformity findings of assessments done at baseline and at different time points within the mentorship period such as inventory control, investigating and documenting corrective actions and the reviewing of L-J charts.

#### Presentations

Presentations on selected topics were made for the entire laboratory team once every week on a fixed day and time. Topics for presentation were based on the nonconformities indentified during the baseline and/or exit assessments. Examples include: corrective action investigation and reporting, external quality assurance (EQA), plotting and review of L-J charts, inventory control at facility level and competency assessments.

#### Staff meetings

Regularly scheduled laboratory meetings were held during the mentorship periods. During these meetings, the mentor provided coaching and provided advice on issues arising from the laboratory. These meetings were led by the laboratory supervisor and were an opportunity to reinforce the utility of staff meetings for communication and the need to document discussions and the resulting actions.

The aforementioned mentoring methods were used together in all four laboratories and none were individually assessed for efficacy.

### Measuring laboratory progress

Standardised measures of performance to gauge laboratory progress and mentoring effectiveness was conducted at specific time points within the 10 week mentorship period: at initial baseline, at the end of the first six week engagement, and at both the start and end of the second four week engagement (see Figure 1).

For the three district laboratories, the WHO-AFRO SLIPTA checklist was used to collect and measure performance. The mentor, who was a WHO-AFRO trained assessor, made all measurements. The checklist, based on the ISO 15189:2007(E) standard and the CLSI guideline GP26-A3,^10^ quantitatively measures adherence to accreditation requirements for quality and competency. The scored checklist (total possible score is 250) is divided into 12 sections that cover the 12 Quality System Essentials (QSE)^11^ (Table 4). The scoring allows the checklist to assign the laboratory a zero to five star rating. The WHO-AFRO SLIPTA checklist star rating was as follows: 0–137: 0 stars, 138–160: 1 star, 161–185: 2 stars; 186–211: 3 stars, 212–236: 4 stars and 237–250: 5 stars (Table 4).

**TABLE 4a:** Summary of WHO AFRO SLIPTA checklist that covers the 12 Quality System Essentials and the weighted marks of each section out of the 250 total points.

Section		Total points	Assessed score^†^
1.	Documents and records	25	
2.	Management reviews	12	
3.	Organisation and personnel	20	
4.	Client management and customer service	8	
5.	Equipment	30	
6.	Internal audit	10	
7.	Purchasing and inventory	30	
8.	Information management	14	
9.	Process control and internal and external quality assessment	43	
10.	Corrective action	8	
11.	Occurrence/incident management and process improvement	10	
12.	Facilities and safety	40	
**Total score**		**250**	

† The assessed score for each section would be placed in this column.

**TABLE 4b:** Summary of WHO AFRO SLIPTA checklist that covers the 12 Quality System Essentials and the weighted marks of each section out of the 250 total points.

Number of stars	Points range	Percentage range (%)
0	0–137	< 55
1	138–160	55–64
2	161–185	65–74
3	186–211	75–84
4	212–236	85–94
5	237–250	> 95

For the Central Laboratory sections of chemistry, haematology, CD4 count and cytology, mentorship was conducted and progress monitored for each section individually. A section-specific checklist was developed, covering the 12 Quality System Essentials and used to monitor progress for five different sections.

## Data analysis

Data on laboratory performance was measured using the WHO-AFRO SLIPTA checklist for the three district laboratories and the section-specific checklists for QE II Central Laboratory. Checklist scores were analysed with the paired *t*-test to compare baseline performance with performance after six weeks of mentorship, at the start of the second mentorship period, and at the end of the 10 weeks of mentorship.

## Results

For the three district laboratories, the average baseline score was 107.7 out of a possible 250 (43.6%) (range 109–138) (see Table 5). At baseline, all three laboratories scored zero stars on the WHO-AFRO SLIPTA star scale. After six weeks of mentorship, the average score was 136.3 (54.5%) (range 119–146). Whilst the scores were numerically not significantly higher than the baseline (*p* = 0.25), two of the three laboratories had shifted to a one star status on WHO-AFRO SLIPTA scale by six weeks. The scores at the start of the second mentorship engagement had remained stable (average 160.5; *p* = 0.096) and the one star status of the two laboratories was maintained. However, after an additional four weeks of mentorship, the average score was 182.3 (range 165–195), a significant increase of 74.7 points on average over the baseline scores (*p* = 0.034). Two laboratories had achieved two stars and the third had achieved a three star status.

**TABLE 5:** Assessment scores at specific time points during the mentorship periods at three district laboratories in Maseru, Lesotho.

Laboratory name	Baseline	End of six weeks mentorship	Start of 2nd mentor period	End of ten weeks mentorship
Mafeteng	109	146	163	198
Motebang	76	119	158	165
Scott	138	144	-	184

**FIGURE 2:**
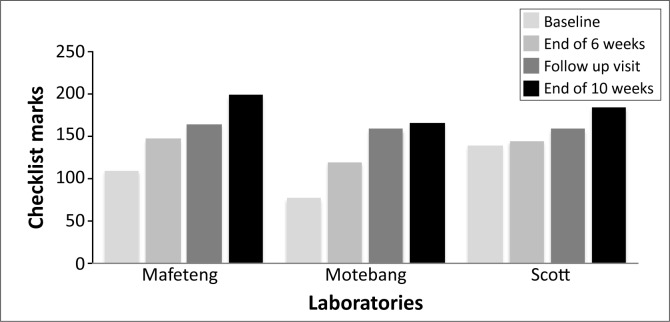
Performance of the three district laboratories at baseline, end of six weeks, beginning of four weeks follow-up visit and at 10 weeks using the SLIPTA checklist.

We illustrate the progress of the three district laboratories at the baseline, at the end of six weeks, the beginning of the four weeks of engagement and at 10 weeks (see Figure 2).

**FIGURE 3:**
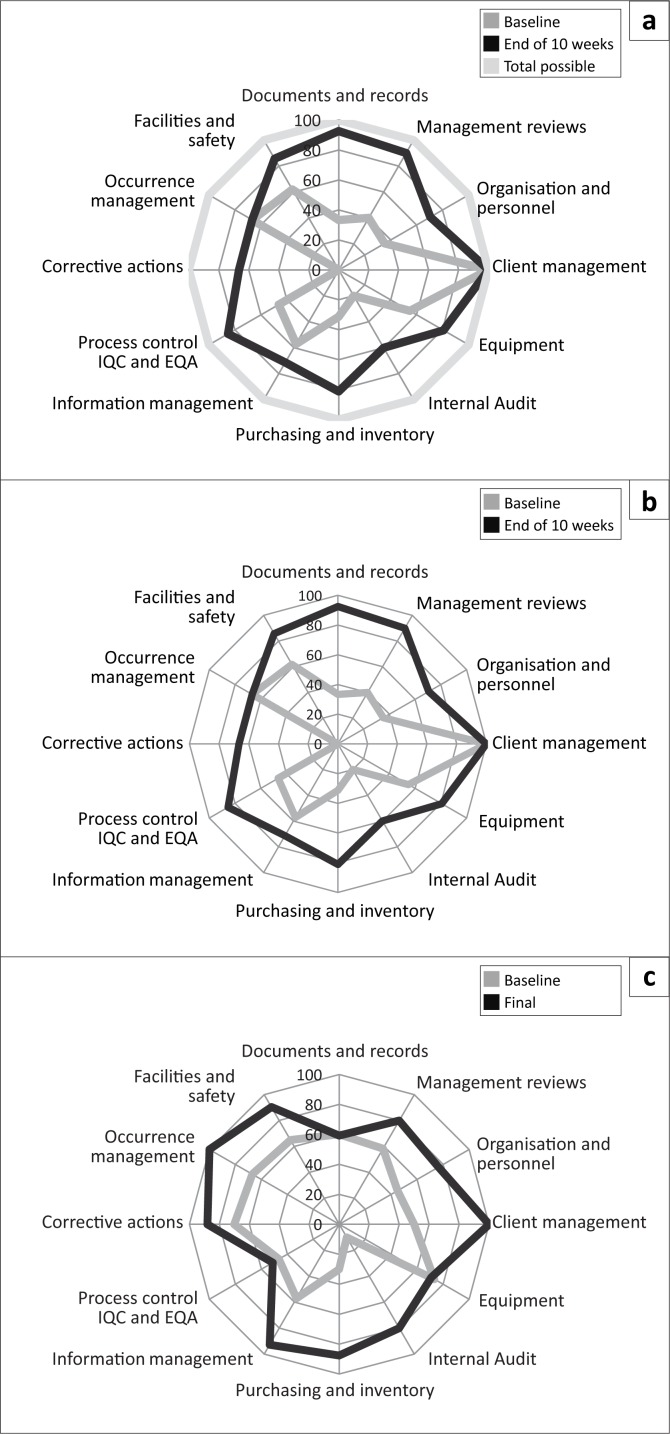
Performance of (a) Mafeteng District Laboratory, (b) Motebang District Laboratory and (c) Scott District Laboratory on the 12 sections of the SLIPTA checklist over 10 weeks of mentorship.

The WHO-AFRO SLIPTA checklist has 12 sections, each with a different weight in marks, all adding up to 250. The progress shown by the 3 laboratories across the 12 sections of the SLIPTA checklists is also illustrated (see Figure 3a, b and c).

**FIGURE 4:**
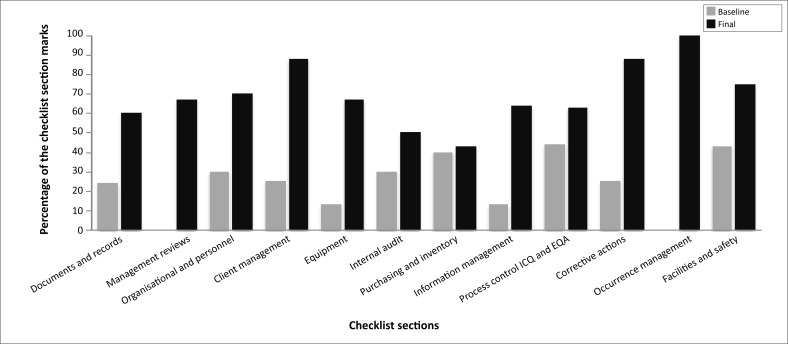
Average performance (based on the WHO-AFRO SLIPTA checklist) of the three district laboratories over the 10 weeks mentorship measured at the four time points during the study: (1) initial baseline, (2) exit after first six weeks mentorship, (3) start of second mentorship period and (4) at the end of the 10 weeks of mentorship. Average marks of the three laboratories are expressed as a percentage of the weighted total for each of the 12 sections of the checklist.

After 10 weeks of mentorship, all three district laboratories improved their scores in client management from an average of 58% to 100% and achieved more than 80% scores in management reviews, facilities and safety and occurrence management from baseline scores of 33%, 57% and 56%, respectively. Average scores for implementation of corrective actions improved from 25% to 67%. Management reviews and internal audits showed the highest percentage change, 46% and 43% respectively (see Figure 4).

Improvements in the areas of management reviews, internal audits and corrective actions were important, as these areas are critical in the continual improvement process. Through management reviews and internal audits the laboratory is able to continuously review and self-evaluate its quality management system. The identified opportunities for improvement need the laboratory to have the ability to implement and document corrective actions.

For the five sections of the QE II Central Laboratory, the section specific checklist was scored in percentages. At five weeks, the average score was 58.8%, which was significantly different from the baseline score of 44% (*p* = 0.017) (Table 6).

**TABLE 6:** Assessment scores at specific time points during the mentorship periods at Queen Elizabeth II Central Laboratory.

Laboratory section	Baseline	End of five weeks mentorship	Start of 2nd mentor period	End of ten weeks mentorship
CD4 Laboratory 1	44	61	-	64
CD4 Laboratory 2	13	48	-	50
FBC Section	44	70	-	54
Cytology laboratory	24	48	-	-
Chemistry	45	59	-	67

FBC, full blood count.

The section-specific checklist was out of total possible marks of 250.

## Discussion

### Outline of results

The mentorship programme in this study was associated with measurable improvement in the laboratory performance, as measured by the WHO AFRO SLIPTA assessment tool. The mentored district laboratories moved from zero stars on the WHO-AFRO SLIPTA scale to two or three out of a possible five stars (average increase of 74.7% from baseline score; *p* = 0.034). This represents a substantial improvement achieved over a relatively short time with a moderate investment of mentor time (10 weeks of mentorship over a six month period). Based on the findings of this study, mentorship may be an effective mechanism to assist progress towards accreditation. We believe that mentorship is complementary to and can be implemented in conjunction with other management training programmes such as SLMTA for optimal impact (SLMTA is a task based training launched by WHO in 2009 that train laboratory managers in the implementation of the quality management system requirements of the WHO AFRO SLIPTA process and eventual international accreditation^11^).

Client service and customer satisfaction improved faster than other sections by reaching, on average, 100% by 10 weeks. At the beginning of the mentorship, all four laboratories had laboratory hand-book, suggestion boxes and appropriately trained staff. Mentorship built on these areas by assisting with conducting of customer surveys, reviewing findings and implementing improvements. Having specific individuals tasked with safety, contributed to improvements to average mark of over 80% after 10 weeks as these individual received targeted mentorship. In the mentor work schedules, internal audits and management reviews appeared in the first six weeks engagement and were mentored on throughout the 10 weeks. This contributed to the observed improvements of an average of over 80%.

The support from the Laboratory Directorate and the Quality Assurance Unit (QAU) of the Ministry of Health of Lesotho contributed significantly to the improvements observed. The laboratory director introduced the mentor to the hospital managers and the laboratories. It was important for the mentor to be seen as an extension of the Ministry of Health. The mentor was allowed direct access to the Laboratory Director with regular meetings and reporting. The QAU reviewed and authorised the quality documents that were introduced at these four laboratories.

In addition, the mentor had access to strong logistical support for transport, accommodation and communication ensuring the smooth operation of the mentorship. This allowed the mentor to concentrate on mentoring.

### Practical implications

In this study, the district laboratories did not show significant improvement before completing 10 weeks of mentorship. This was likely because early improvement initiatives took time to implement. During the initial engagement, the mentor schedules prioritised areas that allowed the laboratory to set the basis for resolving nonconformities. For example, initial engagement prioritised identification of the areas that needed standard operating procedures (SOPs), training on how to write SOPs and then to have staff start to develop and implement these SOPs. In terms of assessment scores, improvement would only be noted when these SOPs were in place and being implemented. In a similar way, training on identification, investigation and documenting of corrective actions was done during the early phases of mentorship. Corrective actions form the basis of resolving all nonconformities encountered in the laboratory such as Internal Quality Control (IQC), EQA and customer complains to stock management. Once the staff are trained and the system put in place, the improvements in terms of assessment scores will be evident when these improvements are constantly being implemented.

The limited measurable improvement before 10 weeks was evidence that mentorship can only be effective if conducted over a sustained period of time. At the QE II Central Laboratory where sections had smaller staff complements and smaller test menus than district laboratories, improvements were reflected faster within five weeks.

### Limitations of the study

One of the limitations of this study was the absence of control laboratories that were followed over the same period of time. Accordingly it was not possible to compare the improvements with laboratories that did not receive mentorship. Whilst it is possible that the improvements observed in this study were random or due to secular influences, we believe that this is unlikely given the magnitude of the observed change and the fact that no other training or laboratory management initiatives were implemented at these sites at the time of the study. It also remains to be determined how cost-effective this type of mentorship is when compared with other initiatives to assist laboratories in achieving accreditation.

### Recomendations

To ensure continued monitoring of laboratory progress beyond the mentorship period, it is recommended that assessments be conducted every six months with remote assistance where needed. Mentorship programmes should also be aligned with the laboratory accreditation goals and the objectives of the Ministry of Health. Findings from mentorship should also inform the national quality assurance system. As we have shown in this study (see Figure 3), mentoring can yield many service level benefits, but a number of critical areas may require action on systems that are coordinated at national management level, often beyond the level of influence of individual laboratories. Hence, parallel national strengthening initiatives for areas such as service and maintenance of instruments, supply chain, proficiency testing and routine assessment of laboratory performance are essential.

## Conclusion

The use of standard tools for assessments allowed comparison across laboratories and aligned improvements to the WHO-AFRO SLIPTA process. Data collection at specific points allowed the mentor to track progress and gauge how much time should be spent on site in order to achieve significant improvements.

Embedding the mentor within the daily routines of the laboratory reduced the supervisory nature of the mentorship and encouraged peer-to-peer relationships to develop between the mentor and the laboratory mentees. This may have created an environment for the mentor to foster positive changes in the laboratory.

Based on these findings we believe that this laboratory mentorship model provides an opportunity for rapid laboratory quality improvement. The method is less dedicated to training in specific technical skills but more focused on training laboratory staff on the management of laboratories related to accreditation. By being iterative and workplace-based, it has an advantage over purely didactic based training in that it provides an extension of learning beyond the classroom to the workplace. This approach seeks to translate knowledge gained into daily practices and hence, re-enforces behaviour change. We recommend that mentorship become a key component of laboratory quality improvement programmes, especially those targeting accreditation.
